# Mitochondrial Respiratory Dysfunction Is Not Correlated With Mitochondrial Genotype in Premature Aging Mice

**DOI:** 10.1111/acel.70085

**Published:** 2025-05-02

**Authors:** Hiroaki Tamashiro, Kaori Ishikawa, Koichi Sadotomo, Emi Ogasawara, Kazuto Nakada

**Affiliations:** ^1^ Graduate School of Science and Technology University of Tsukuba Ibaraki Japan; ^2^ Institute of Life and Environmental Sciences University of Tsukuba Ibaraki Japan; ^3^ Department of Biological Sciences Graduate School of Science, Osaka University Osaka Japan

**Keywords:** aging, mitochondria, mitochondrial DNA

## Abstract

mtDNA mutator mice (*Polg* ^mut/mut^ mice) have reinforced the mitochondrial theory of aging. These mice accumulate multiple mutations in mtDNA with age due to a homozygous proofreading‐deficient mutation in mtDNA polymerase gamma (*Polg*), resulting in mitochondrial respiratory dysfunction and premature aging phenotypes. However, whether the accumulation of multiple mutations in *Polg* ^mut/mut^ mice induces mitochondrial respiratory dysfunction remains unclear. Here, we determined the accurate mtDNA genotype, including the frequency of total mutations and the number of non‐synonymous substitutions and pathogenic mutations, using next‐generation sequencing in the progeny of all three genotypes obtained from the mating of heterozygous mtDNA mutator mice (*Polg* ^+/mut^ mice) and examined their correlation with mitochondrial respiratory activity. Although *Polg* ^+/mut^ mice showed equivalent mtDNA genotype to *Polg* ^+/+^ (wild‐type) mice, the mitochondrial respiratory activity in the *Polg* ^+/mut^ mice was mildly reduced. To further investigate the causal relationship between mtDNA genotype and mitochondrial respiratory activity, we experimentally varied the mtDNA genotype in *Polg* mice. However, mitochondrial respiratory activity was mildly reduced in *Polg* ^+/mut^ mice and severely reduced in *Polg* ^mut/mut^ mice, regardless of the mtDNA genotype. Moreover, by varying the mtDNA genotype, some *Polg* ^+/+^ mice showed mtDNA genotype equivalent to those of *Polg* ^mut/mut^ mice, but mitochondrial respiratory activity in *Polg* ^+/+^ mice was normal. These results indicate that the mitochondrial respiratory dysfunction observed in mice with proofreading‐deficient mutation in *Polg* is correlated with the nuclear genotype of *Polg* rather than the mtDNA genotype. Thus, the mitochondrial theory of aging in *Polg* ^mut/mut^ mice needs further re‐examination.

## Graphical abstract

The causal relationship between random mtDNA mutations and reduced mitochondrial respiratory activity in premature aging *Polg* ^mut/mut^ mice remains unclear. This study shows that the abnormal mitochondrial function in *Polg* ^mut/mut^ mice is more closely associated with the nuclear *Polg* genotype than with the mtDNA genotype.

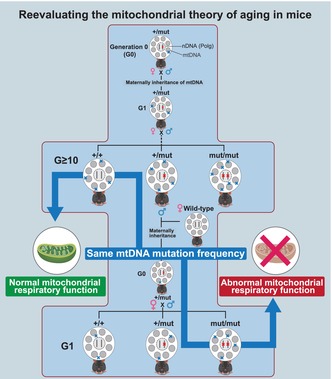

## 1 Introduction

Point mutations and deletions in mtDNA can reduce the translation of respiratory complex subunits, leading to mitochondrial respiratory dysfunction and mitochondrial diseases, as well as other diseases such as diabetes and cancer, and contributing to aging (Wallace 1999). Because mtDNA is 5–10 times more prone to accumulating mutations than nuclear DNA (Ames et al. 1993; Brown et al. 1979), the mitochondrial theory of aging has been proposed, which suggests that the accumulation of multiple mutations in mtDNA with age induces mitochondrial respiratory dysfunction, ultimately leading to aging (Harman 1956). mtDNA mutator mice (*Polg* ^mut/mut^ mice) with a homozygous proofreading‐deficient mutation in the *Polg* gene accumulate multiple mutations in mtDNA, resulting in mitochondrial respiratory dysfunction and premature aging phenotypes (Kujoth et al. 2005; Trifunovic et al. 2004). These mice have been considered model organisms supporting the mitochondrial theory of aging. However, previous studies have reported that multiple deletions and specific pathogenic mutations in mtDNA do not result in premature aging phenotypes (Fan et al. 2008; Hashizume et al. 2012; Inoue et al. 2000; Kasahara et al. 2006; Lin et al. 2012; Shimizu et al. 2014; Tani et al. 2022; Tyynismaa et al. 2005; Yokota et al. 2010; Zhang et al. 2025). In addition to mitochondrial respiratory dysfunction, somatic stem cell dysfunction (Ahlqvist et al. 2012; Hämäläinen et al. 2015) and immunometabolism dysfunction (Lei et al. 2021) have been proposed as factors for premature aging in *Polg* ^mut/mut^ mice. However, the causal relationship between random point mutations accumulating in mtDNA and mitochondrial respiratory dysfunction in *Polg* ^mut/mut^ mice remains unclear.

Previous studies have reported that neither random point mutations nor deletions in mtDNA are pathogenic (Edgar et al. 2009; Vermulst et al. 2007), and the accumulation of random non‐synonymous substitutions in mtDNA, among other things, may induce mitochondrial respiratory dysfunction in *Polg* ^mut/mut^ mice (Edgar et al. 2009). However, the pathogenicity of random non‐synonymous substitutions in *Polg* ^mut/mut^ mice may be suppressed due to the presence of inter‐mitochondrial complementation and a threshold effect (Nakada et al. 2001; Ono et al. 2001). Thus, it has been hypothesized that mtDNA molecules with a low frequency of pathogenic mutations clonally expand above a threshold, leading to mitochondrial respiratory dysfunction in *Polg* ^mut/mut^ mice (Trifunovic et al. 2004; Vermulst et al. 2008). However, to date, experimental evidence for clonal expansion of mtDNA molecules with pathogenic mutations causing mitochondrial respiratory dysfunction in these mice is lacking.

Therefore, the causal relationship between the accumulation of random point mutations in mtDNA and mitochondrial respiratory dysfunction, as proposed in the mitochondrial theory of aging, remains unclear. Contradicting this theory, a previous study has suggested that the accumulation of random point mutations in mtDNA cannot be pathogenic and has focused on not only *Polg* ^mut/mut^ mice but also on *Polg* ^+/mut^ mice with a heterozygous proofreading‐deficient mutation in *Polg* (Vermulst et al. 2007). However, no detailed investigations have explored the correlation between mtDNA genotype (including *Polg* ^+/mut^ mice), such as the frequency of total mutations, the number of non‐synonymous substitutions, and pathogenic mutations, and mitochondrial respiratory dysfunction.

Accurate detection of mtDNA mutations is essential for examining the causal relationship between mtDNA genotype and reduced mitochondrial respiratory function in Polg mice. However, PCR amplification of partial regions of mtDNA or full‐length mtDNA for DNA sequencing (Ma et al. 2018; Ni et al. 2015) could amplify nuclear mitochondrial segments (NUMTs) (Goios et al. 2009, 2008) and fragmented mtDNA, which are increased in *Polg* ^mut/mut^ mice (Edgar et al. 2009), making accurate detection of mtDNA challenging. In fact, previous reports on mtDNA mutation frequency in *Polg* ^mut/mut^ mice, compared with wild‐type mice, vary from 3 to 8‐fold (Kujoth et al. 2005; Trifunovic et al. 2004) to 2500‐fold (Vermulst et al. 2007) increase.

In this study, we investigated the causal relationship between mtDNA genotype and mitochondrial respiratory function in *Polg* ^+/mut^ mice, in addition to *Polg* ^+/+^ and *Polg* ^mut/mut^ mice. To accurately detect mtDNA mutations, we used enriched circular mtDNA from organs of *Polg* mice without PCR amplification for next‐generation sequencing (Bagge et al. 2020). Furthermore, to examine this relationship in more detail, we generated *Polg* mice with the same nuclear genotype as *Polg* but varying mtDNA genotype and compared their mitochondrial respiratory activity. Our findings demonstrate that mitochondrial respiratory dysfunction is not due to mtDNA genotype in *Polg* ^mut/mut^ mice, suggesting that there is no causal relationship between the accumulation of random point mutations in mtDNA and mitochondrial respiratory dysfunction in mice, in contrast to the mitochondrial theory of aging.

## 2 Materials and Methods

### 2.1 G ≥ 10 and G1 *Polg* Mice

Based on prior research (Trifunovic et al. 2004; Kujoth et al. 2005), we generated *Polg* ^+/mut^ and *Polg* ^mut/mut^ mice with a C57BL/6J nuclear background (Mito et al. 2013) as the 0th generation (G0) of the mouse strain in our previous study. G0 *Polg* ^+/mut^ mice were crossed to generate G1 *Polg* mice, and G1 *Polg* ^+/mut^ mice were then crossed to generate G2 *Polg* mice. Thus, we utilized G ≥ 10 (including G10–G13) *Polg* ^+/+^, *Polg* ^+/mut^, and *Polg* ^mut/mut^ mice in this study. In the strains maintained by crossing *Polg* ^+/mut^ mice, it was assumed that random point mutations occurring in mtDNA during oocyte maturation are maternally inherited, leading to the accumulation of random point mutations in mtDNA through the maternal germ line in G ≥ 10 *Polg* mice (Ross et al. 2013; Yang et al. 2020). To eliminate maternally inherited random point mutations in mtDNA of G ≥ 10 *Polg* mice, G0 *Polg* ^+/mut^ mice were generated by crossing male G ≥ 10 *Polg* ^+/mut^ mice with female B6 (wild‐type) mice. Then, by crossing G0 *Polg* ^+/mut^ mice, we obtained G1 *Polg* ^+/+^, *Polg* ^+/mut^, and *Polg* ^mut/mut^ mice (Figure 2A), which were used in this study. Male G ≥ 10 and G1 *Polg* mice at 8–10 months of age were used for the analysis. All animal experiments were approved by the Institutional Animal Care and Use Committee of the University of Tsukuba.

### 2.2 mtDNA Enrichment From Mouse Tissue

To accurately detect mutations in mtDNA, we enriched circular mtDNA by treating total DNA obtained from mouse organs with Exonuclease V (Exo V), which degrades linear DNA, based on a previous report (Bagge et al. 2020). The restriction enzyme reaction solution consisted of 2 μL of Exo V (#M0345S, NEB), 10 μL of NEB buffer 4 (10×), and 10 μL of ATP (10 mM). Approximately 5000 ng of total DNA was added to the solution, adjusted to a final volume of 100 μL with distilled water, and incubated at 37°C overnight. The solution was further treated with 2 μL of NEB buffer 4 (10×), 8 μL of ATP (10 mM), 8 μL of Exo V, and 2 μL of distilled water at 37°C overnight to fully degrade the linear DNA. The remaining circular mtDNA was extracted using 0.4 vol AMPure XP beads (#A63881, Beckman).

### 2.3 Next‐Generation Sequencing of mtDNA

Following library preparation from enriched mtDNA using the TruePrep DNA library prep kit (Vazyme # TD501‐503), deep sequencing was conducted using Hiseq 2000 (Illumina), with chrM of the UCSC mm10 as a reference sequence. Total read numbers, average depth, and coverage rate for each sample are indicated in Table S1. mtDNA mutation frequency (%) indicates the probability that a mutation occurs in each base of mtDNA (mtDNA mutation frequency = number of bases differing from the reference sequence × 100/total number of bases). Non‐synonymous substitutions and pathogenic mutations were quantified for positions where the mutation frequency exceeded 1%. Alignments for nucleic acid and amino acid sequences from mice and humans were performed for every gene and region using T‐COFFEE.

### 2.4 COX/SDH Staining

COX/SDH staining is a histochemical method used to assess mitochondrial respiratory activity (Trifunovic et al. 2004; Wai et al. 2008; Wang et al. 1999), with COX staining for detecting the activity of respiratory enzyme complex IV (COX) and SDH staining for detecting the activity of respiratory enzyme complex II (SDH). When the mitochondrial respiratory function is reduced, COX staining (brown) based on the activity of respiratory enzyme complex IV is reduced, and SDH compensates by facilitating redox reactions, resulting in blue‐stained cells (COX−/SDH+). In cells with normal mitochondrial respiratory function, the activity of respiratory enzyme complex IV is maintained, resulting in brown cells (COX+/SDH−).

Using these techniques, mitochondrial respiratory activity was assessed in fresh‐frozen sections (10 μm thick) prepared from the heart, kidney, and soleus of G ≥ 10 and G1 *Polg* mice. The cryosections were reacted with 2 mg/mL diaminobenzidine tetrahydrochloride (DOJINDO, #349–00903) in 0.1 M acetate buffer (pH 5.5) containing 0.1% MnCl_2_ and 0.1% H_2_O_2_, followed by incubation with 1 mg/mL of NBT (Wako, #144–01993) in 0.2 M phosphate buffer (pH 7.4) with sodium succinate. Images were acquired using a microscope camera (DFC310FC, Leica) after observation with a microscope (DMRE, Leica). To quantify cells with reduced mitochondrial respiratory activity, the percentage of COX−/SDH+ cells in each organ was calculated based on the stained images.

### 2.5 Statistical Analysis

The sample size of each test and statistical method used for data analysis are stated in each figure legend. Statistical analyses were conducted using R and RStudio. A *p*‐value of less than 0.05 was considered to indicate statistically significant differences between samples.

## 3 Results

### 3.1 mtDNA Mutation Frequency Is Not Correlated With Mitochondrial Respiratory Activity in G ≥ 10 *Polg* Mice

**FIGURE 1:**
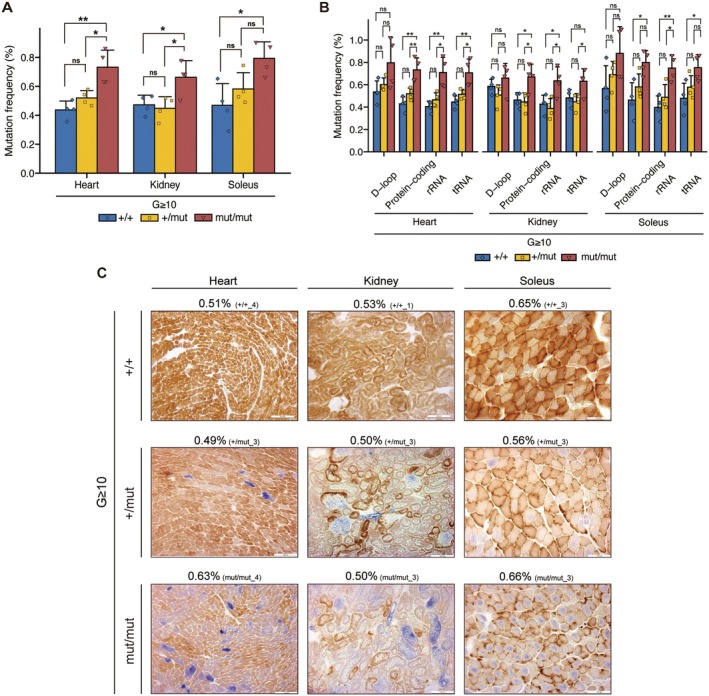
Comparison of mtDNA mutation frequency and mitochondrial respiratory activity in organs of G ≥ 10 *Polg* ^+/+^, *Polg* ^+/mut^, and *Polg* ^mut/mut^ mice. (A) mtDNA mutation frequency in the heart, kidney, and soleus of G ≥ 10 *Polg* ^+/+^, *Polg* ^+/mut^, and *Polg* ^mut/mut^ mice (*n* = 4 for each group). Data are presented as mean ± SD. *p < 0.05, **p < 0.01 (Tukey–Kramer test). (B) mtDNA mutation frequency in each gene region of mtDNA in the organs of G ≥ 10 *Polg* ^+/+^, *Polg* ^+/mut^, and *Polg* ^mut/mut^ mice (*n* = 4, each group). Data are presented as mean ± SD. *p < 0.05, **p < 0.01 (Tukey–Kramer test). (C) COX/SDH staining results in each organ (heart, kidney, soleus) of G ≥ 10 *Polg* ^+/+^, *Polg* ^+/mut^, and *Polg* ^mut/mut^ mice. Numbers above the stained images indicate mtDNA mutation frequency in each organ and the Mouse ID identifying each mouse. Scale bars, 100 μm.

To investigate the correlation between mtDNA genotype and mitochondrial respiratory function, we enriched mtDNA in the heart, kidney, and soleus of G ≥ 10 *Polg* mice, followed by next‐generation sequencing (see Material and Methods). Our analysis revealed that, compared with G ≥ 10 *Polg* ^+/+^ mice, G ≥ 10 *Polg* ^mut/mut^ mice exhibited a significantly a higher mutation accumulation in mtDNA, with the mutation frequency reaching up to approximately 1.7 times higher (Figure 1A). Conversely, G ≥ 10 *Polg* ^+/mut^ mice showed no statistically significant difference in the mutation frequency compared with *Polg* ^+/+^ mice across all organs (Figure 1A). We further examined whether mtDNA mutations caused by the proofreading deficiency of *Polg* were influenced by gene function (Figure 1B). The results indicated that G ≥ 10 *Polg* ^mut/mut^ mice exhibited a higher mutation frequency than G ≥ 10 *Polg* ^+/+^ mice across all gene regions, with significant differences in all gene regions except for the D‐loop (Figure 1B). In contrast, compared with G ≥ 10 *Polg* ^+/+^ mice, G ≥ 10 *Polg* ^+/mut^ mice exhibited a similar mutation frequency in all gene regions (Figure 1B). These results suggest that random point mutations significantly accumulate in the mtDNA of mice only when the proofreading capacity of *Polg* is homozygously defective, with mutations occurring randomly across all gene regions of the mtDNA.

Next, to examine the correlation between the mutation frequency and mitochondrial respiratory function, we assessed mitochondrial respiratory activity in each organ of G ≥ 10 *Polg* mice by COX/SDH staining. Our results revealed that mitochondrial respiratory activity was mildly reduced in *Polg* ^+/mut^ mice and severely reduced in *Polg* ^mut/mut^ mice across all organs (heart, kidney, and soleus) (Figure S1A–C). Notably, some G ≥ 10 *Polg* mice exhibited similar mutation frequency across the organs despite having different nuclear *Polg* genotypes (Figure 1C; red frame in Figure S1A–C). Regardless of the mutation frequency, mitochondrial respiratory activity in these organs was mildly reduced in *Polg* ^+/mut^ mice and severely reduced in *Polg* ^mut/mut^ mice (Figure 1C; red frame in Figure S1A–C). For example, in the soleus, the mutation frequency of *Polg* ^+/mut^ mice was 0.56%, which was lower than that of *Polg* ^+/+^ mice; however, mitochondrial respiratory activity in *Polg* ^+/mut^ mice was mildly reduced (Figure 1C). Similarly, although the mutation frequency of *Polg* ^mut/mut^ mice was comparable to that of *Polg* ^+/+^ mice (0.66%), their mitochondrial respiratory activity was severely reduced (Figure 1C).

### 3.2 Generation of Mice With Experimentally Reduced mtDNA Mutation Frequency

To further investigate the causal relationship between random point mutations in mtDNA and mitochondrial respiratory function, we attempted to vary mtDNA genotype in *Polg* mice. *Polg* mice are assumed to accumulate random point mutations in mtDNA through the maternal germline (see Material and Methods). Indeed, mtDNA mutations accumulating in the organs of G ≥ 10 *Polg* mice showed a high mutation frequency (Figure S2A–C), which remained consistent across all organs in each mouse (Figure S2D–I). These results indicate that random point mutations in mtDNA occurring in the oocyte are inherited maternally and accumulate at similar levels across organs in mice.

**FIGURE 2:**
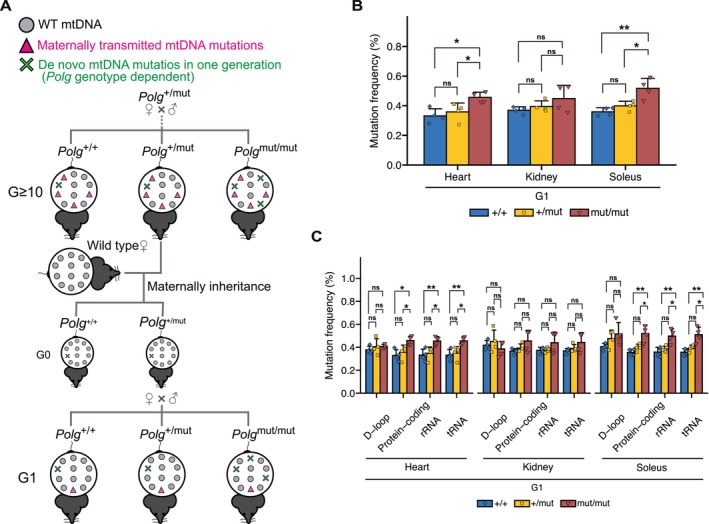
Generation of G1 *Polg* ^+/+^, *Polg* ^+/mut^, and *Polg* ^mut/mut^ mice. (A)To create G0 *Polg* ^+/mut^ mice with wild‐type mtDNA, male G ≥ 10 *Polg* ^+/mut^ mice were crossed with female wild‐type mice. These G0 *Polg* ^+/mut^ mice were then crossed to generate G1 *Polg* ^+/+^, *Polg* ^+/mut^, and *Polg* ^mut/mut^ mice. (B) mtDNA mutation frequency in the heart, kidney, and soleus of G1 *Polg* ^+/+^, *Polg* ^+/mut^, and *Polg* ^mut/mut^ mice (*n* = 4, each group). Data are presented as mean ± SD. *p < 0.05, **p < 0.01 (Tukey–Kramer test). (C) mtDNA mutation frequency by gene region in the heart, kidney, and soleus of G1 *Polg* ^+/+^, *Polg* ^+/mut^, and *Polg* ^mut/mut^ mice (*n* = 4, each group). Data are presented as mean ± SD. *p < 0.05, **p < 0.01 (Tukey–Kramer test).

Thus, to eliminate random point mutations in mtDNA accumulated through the germline in G ≥ 10 *Polg* mice, we generated G1 *Polg* (Figure 2A, see Material and Methods) and conducted mtDNA analysis using next‐generation sequencing. The accumulation of mtDNA mutations with a higher frequency observed in G ≥ 10 *Polg* mice (Figure S2A–C) was rare in G1 *Polg* mice (Figure S3A–C), and a mutation frequency in G1 *Polg* mice was lower than that in G ≥ 10 *Polg* mice (Figures 1A and 2B). Furthermore, G ≥ 10 *Polg* mice exhibited greater variation in mutation frequency compared to G1 *Polg* mice (Figures 1A and 2B). This variability may reflect differences in the number of generations, as G ≥ 10 *Polg* mice include G10‐13 *Polg* mice. In G1 *Polg* mice, a mutation frequency was significantly higher in the heart and soleus of *Polg* ^mut/mut^ mice than in those of *Polg* ^+/+^ mice, whereas the mutation frequency of *Polg* ^+/mut^ mice was approximately equal to that of *Polg* ^+/+^ mice (Figure 2B). This pattern was further confirmed by analyzing a mutation frequency across gene regions, revealing that *Polg* ^mut/mut^ mice accumulated significantly more mtDNA mutations than *Polg* ^+/+^ mice, except for the D‐loop region, whereas *Polg* ^+/mut^ mice showed a similar mutation frequency to *Polg* ^+/+^ mice across all regions (Figure 2C).

### 3.3 Non‐Synonymous Substitution and Pathogenic Mutations Accumulate in mtDNA Through Maternal Germline in *Polg* Mice

A previous study has demonstrated purifying selection against non‐synonymous substitution in mtDNA in the germline (Stewart et al. 2008). This finding suggests that random point mutations in mtDNA accumulated through the germline in G ≥ 10 *Polg* mice may be neutral point mutations, unlike somatic mutations in mtDNA. If so, variation in mtDNA genotype through the germline is not an appropriate approach to examine the causal relationship between random point mutations in mtDNA and mitochondrial respiratory dysfunction, which is the basis of the mitochondrial theory of aging.

**FIGURE 3:**
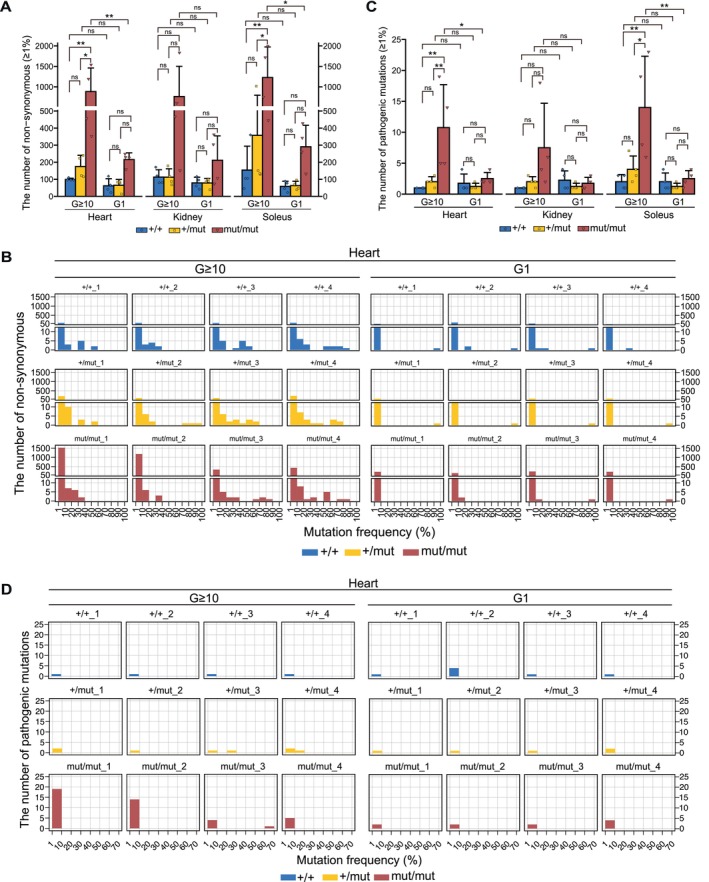
Number of non‐synonymous substitutions and pathogenic mutations in G ≥ 10 and G1 *Polg* ^+/+^, *Polg* ^+/mut^, and *Polg* ^mut/mut^ mice. (A) Mean number of non‐synonymous substitutions with a mutation frequency of more than 1% in the organs of G ≥ 10 and G1 *Polg* ^+/+^, *Polg* ^+/mut^, and *Polg* ^mut/mut^ mice (*n* = 4, each group). Data are reported as mean ± SD. *p < 0.05, **p < 0.01 (Tukey–Kramer test). (B) Histogram of the number of non‐synonymous substitutions in the heart of individual G ≥ 10 and G1 *Polg* ^+/+^, *Polg* ^+/mut^, and *Polg* ^mut/mut^ mice. The x‐axis represents mutation frequency, and the y‐axis represents the number of non‐synonymous substitutions. Mouse IDs are displayed above each histogram. (C) Mean number of pathogenic mutations with a mutation frequency of more than 1% in the organs of G ≥ 10 and G1 *Polg* ^+/+^, *Polg* ^+/mut^, and *Polg* ^mut/mut^ mice (*n* = 4, each group). Data are presented as mean ± SD. *p < 0.05, **p < 0.01 (Tukey–Kramer test). Histogram of the number of pathogenic mutations in the heart of individual G ≥ 10 and G1 *Polg* ^+/+^, *Polg* ^+/mut^, and *Polg* ^mut/mut^ mice. The x‐axis represents mutation frequency, and the y‐axis represents the number of pathogenic mutations. Mouse IDs are displayed above each histogram (corresponding to Mouse No. in Extended Data Figures [Link], [Link]).

Thus, to assess whether the accumulation of non‐synonymous substitutions in mtDNA varied between generations of mouse strains, non‐synonymous substitutions that accumulated in each organ of G ≥ 10 and G1 *Polg* mice at mutation frequency over 1% were quantified. G ≥ 10 *Polg* ^mut/mut^ mice tended to accumulate more non‐synonymous substitutions in mtDNA than G1 *Polg* ^mut/mut^ mice, with significant differences identified in the heart and soleus (Figure 3A). A similar pattern was also observed in *Polg* ^+/+^ mice and *Polg* ^+/mut^ mice without significant differences (Figure 3A). In G ≥ 10 *Polg* mice, mtDNA mutations with a high mutation frequency assumed to be maternally inherited were observed (Figure S2A–C). To determine whether non‐synonymous substitutions are present in these maternally inherited mutations, a mutation frequency of non‐synonymous substitutions in the organs of G ≥ 10 and G1 *Polg* mice was analyzed. Compared with G1 *Polg* mice, G ≥ 10 *Polg* mice accumulated more non‐synonymous substitutions, with more than 10% mutation frequency (Figures 3B and S4A–B). For example, in the heart, G ≥ 10 *Polg* mice accumulated non‐synonymous substitutions with more than 10% mutation frequency, whereas such non‐synonymous substitutions were rare in G1 *Polg* mice (Figure 3B).

When pathogenic mutations accumulate above a threshold in mtDNA molecules, they cause structural abnormalities in the subunits of the respiratory enzyme complex and impaired translational function, resulting in mitochondrial respiratory dysfunction. Thus, we assessed the mutations homologous to confirmed pathogenic mutations in human mtDNA among the mutations that accumulated in G ≥ 10 and G1 *Polg* mice at a mutation frequency over 1%. All confirmed pathogenic mutations (125 in total, last accessed: October 8, 2024) are listed in MITOMAP (https://mitomap.org). G ≥ 10 *Polg* ^+/+^ and *Polg* ^+/mut^ mice accumulated as many pathogenic mutations as G1 *Polg* ^+/+^ and *Polg* ^+/mut^ mice, respectively (Figure 3C). In contrast, G ≥ 10 *Polg* ^mut/mut^ mice accumulated significantly higher pathogenic mutations in mtDNA than G1 *Polg* ^mut/mut^ mice (Figure 3C). Additionally, pathogenic mutations with more than 10% mutation frequency were observed to accumulate in G ≥ 10 *Polg* ^+/mut^ and *Polg* ^mut/mut^ mice, but no such mutations were present in G1 *Polg* mice (Figures 3D and S5A,B). For example, in the heart of a G ≥ 10 *Polg* ^+/mut^ mouse (Mouse ID: +/mut_3) and G ≥ 10 *Polg* ^mut/mut^ mouse (Mouse ID: mut/mut_3), pathogenic mutations with 20%–30% and 60%–70% mutation frequency were observed to accumulate, respectively (Figure 3D).

These results indicate that non‐synonymous substitutions and pathogenic mutations with more than 1% mutation frequency could accumulate in the mtDNA beyond purifying selection in the germline of the crossed *Polg* ^+/mut^ mice.

### 3.4 Reduced Mitochondrial Respiratory Activity Observed in *Polg* Mice Is Not due to mtDNA Mutation Frequency

**FIGURE 4:**
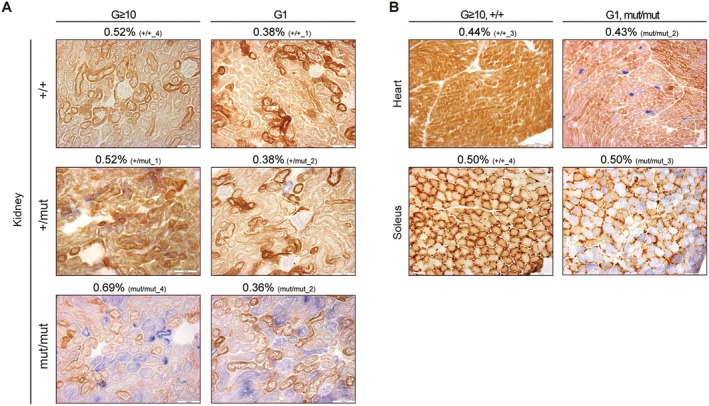
Correlation between mtDNA mutation frequency and mitochondrial respiratory activity in G ≥ 10 and G1 *Polg* mice. (A) COX/SDH staining results for the kidneys of G ≥ 10 and G1 *Polg* ^+/+^, *Polg* ^+/mut^, and *Polg* ^mut/mut^ mice. The numbers above the images represent mtDNA mutation frequency and Mouse ID for each mouse. Scale bars, 100 μm. (B) COX/SDH staining results for the heart and soleus of G ≥ 10 *Polg* ^+/+^ and G1 *Polg* ^mut/mut^ mice. The numbers above the images indicate mtDNA mutation frequency and Mouse ID for each individual mouse. Scale bars, 100 μm.

If the accumulation of random point mutations in mtDNA leads to reduced mitochondrial respiratory function, mitochondrial respiratory activity in G1 *Polg* mice would have improved or normalized compared with that in G ≥ 10 *Polg* mice. To investigate this, we assessed mitochondrial respiratory activity in each organ of G1 *Polg* mice using COX/SDH staining (Figure S6A–C). The results indicated that mitochondrial respiratory activity was mildly reduced in G1 *Polg* ^+/mut^ mice and severely reduced in G1 *Polg* ^mut/mut^ mice (Figure S6A–C). However, a comparison between G ≥ 10 and G1 *Polg* mice revealed that mitochondrial respiratory activity in G1 *Polg* remained equivalent to that in G ≥ 10 *Polg* mice, despite a lower mutation frequency in the G1 *Polg* mice (Figure 4A; blue box in Figures S1A–C and S6A–C). For instance, G1 *Polg* ^mut/mut^ mice exhibited a severe reduction in mitochondrial respiratory activity as G ≥ 10 *Polg* ^mut/mut^ mice, even though the mutation frequency in G1 *Polg* ^mut/mut^ mice was 0.36%, approximately half of the mutation frequency (0.69%) observed in G ≥ 10 *Polg* ^mut/mut^ mice (Figure 4A).

Next, we focused on G1 *Polg* ^mut/mut^ mice that exhibited the same mutation frequency as G ≥ 10 *Polg* ^+/+^ mice (Figures 1A and 2B). If the accumulation of random point mutations in mtDNA causes reduced mitochondrial respiratory activity, G1 *Polg* ^mut/mut^ mice would be expected to exhibit normal mitochondrial respiratory activity, similar to G ≥ 10 *Polg* ^+/+^ mice. However, as mentioned, mitochondrial respiratory activity was severely reduced in G1 *Polg* ^mut/mut^ mice (Figure S6A–C). A detailed comparison of COX/SDH staining between G ≥ 10 *Polg* ^+/+^ and G1 *Polg* ^mut/mut^ mice revealed significant differences in mitochondrial respiratory activity (Figure 4B; green box in Figures S1A–C and S6A–C). For example, in the heart, G1 *Polg* ^mut/mut^ mice exhibited severely reduced mitochondrial respiratory activity (Figure 4B) despite showing the same mutation frequency (0.43%) as G ≥ 10 *Polg* ^+/+^ mice. In the kidney, mitochondrial respiratory activity was severely reduced in G1 *Polg* ^mut/mut^ mice, even though their mutation frequency was 0.36%, which was lower than that of G ≥ 10 *Polg* ^+/+^ mice (0.52%) (Figure 4A).

### 3.5 Reduced Mitochondrial Respiratory Activity Observed in *Polg* Mice Is Not due to the Accumulation of Non‐Synonymous Substitutions and Pathogenic Mutations in mtDNA

**FIGURE 5:**
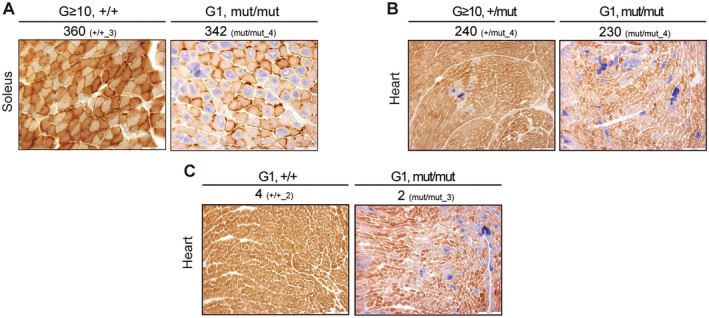
Correlation between non‐synonymous substitutions, pathogenic mutations, and mitochondrial respiratory activity in G ≥ 10 and G1 *Polg* mice. (A) COX/SDH staining comparison for the soleus muscles of G ≥ 10 *Polg* ^+/+^ and G1 *Polg* ^mut/mut^ mice. The numbers above the images show a number of non‐synonymous substitutions and Mouse IDs. Scale bars, 100 μm. The G ≥ 10 *Polg* ^+/+^ image is the same as that in Figure 1C. (B) COX/SDH staining for the heart of G ≥ 10 *Polg* ^+/mut^ and G1 *Polg* ^mut/mut^ mice. The numbers above the images indicate non‐synonymous substitution counts and Mouse ID. Scale bars, 100μm. (C) COX/SDH staining for the heart of G1 *Polg* ^+/+^ and G1 *Polg* ^mut/mut^ mice. The numbers above the images indicate pathogenic mutation counts and Mouse ID. Scale bars, 100 μm.

A previous study has indicated that, contrary to the presence of inter‐mitochondrial complementation and the threshold effect (Nakada et al. 2001; Ono et al. 2001), the random accumulation of non‐synonymous substitutions in mtDNA causes instability of respiratory enzyme complexes, resulting in mitochondrial respiratory dysfunction in *Polg* ^mut/mut^ mice (Edgar et al. 2009). Consequently, we hypothesized that the reduced mitochondrial respiratory activity observed in *Polg* mice was due to non‐synonymous substitutions (quality of mutations that accumulate in mtDNA) rather than mtDNA mutation frequency (number and ratio of all mtDNA mutations that accumulate). To investigate this hypothesis, we examined G ≥ 10 *Polg* ^+/+^ and *Polg* ^+/mut^ mice, which had comparable numbers of non‐synonymous substitutions to G1 *Polg* ^mut/mut^ mice, and assessed their mitochondrial respiratory activity (Figure 5A,B; yellow frame in Figures S1A–C and S6A–C). Our findings revealed that G ≥ 10 *Polg* ^+/+^ mice maintained normal mitochondrial respiratory activity despite accumulating 360 non‐synonymous substitutions, which was more than that observed in G1 *Polg* ^mut/mut^ mice (Figure 5A). G ≥ 10 *Polg* ^+/mut^ mice had fewer non‐synonymous substitutions in the heart than G ≥ 10 *Polg* ^+/+^ mice, but their mitochondrial respiratory activity was mildly reduced (Figure 5B).

As previously discussed, the accumulation of pathogenic mutations in mtDNA causes structural abnormalities in the subunits of the respiratory enzyme complex and impaired translational function, resulting in reduced mitochondrial respiratory activity. A previous study hypothesized that mtDNA molecules with a low frequency of pathogenic mutations clonally expand beyond a threshold with age, resulting in mitochondrial respiratory dysfunction in *Polg* ^mut/mut^ mice (Trifunovic et al. 2004; Vermulst et al. 2008). To examine the causal relationship between mitochondrial respiratory dysfunction and the accumulation of pathogenic mutations in mtDNA, we analyzed G1 *Polg* ^+/+^ mice, which exhibited a comparable number of non‐synonymous substitutions to G1 *Polg* ^mut/mut^ mice, and assessed their mitochondrial respiratory activity (Figure 5C; purple frame in Figures S6A). Despite G1 *Polg* ^+/+^ mice and *Polg* ^mut/mut^ mice accumulating 3 and 2 pathogenic mutations, respectively, only the G1 *Polg* ^mut/mut^ mice exhibited cells with reduced mitochondrial respiratory activity (Figure 5C). This indicates that the reduced mitochondrial respiratory activity in *Polg* ^mut/mut^ mice is not due to the accumulation of pathogenic mutations exceeding 1% mutation frequency. However, a pathogenic mutation (T8393C, homologous to T8993C in humans) with a mutation frequency of 60%–70% accumulated in the protein‐coding (ATP6) region in the heart of a G ≥ 10 *Polg* ^mut/mut^ mouse (Mouse ID: mut/mut_3) (Figure 3D). Thus, it is possible that the reduced mitochondrial respiratory activity observed in this individual (Figure S1A, Mouse ID: mut/mut_3) was caused by the accumulation of this pathogenic mutation.

### 3.6 Reduced Mitochondrial Respiratory Activity in *Polg* Mice Is Not due to the Accumulation of Small Deletions and Insertions in mtDNA

Small insertions, deletions, and point mutations can occur in mtDNA. To investigate whether the accumulation of small deletions and insertions contributes to reduced mitochondrial respiratory activity in *Polg* mice, we analyzed their frequency in different genotypes.

Our results showed that the frequency of small deletions and insertions in G ≥ 10 and G1 *Polg* ^mut/mut^ mice tended to be higher compared to that in G ≥ 10 and G1 *Polg* ^+/+^ mice, respectively (Figure S7A,B). In contrast, the frequency of small deletions and insertions in G ≥ 10 and G1 *Polg* ^+/mut^ mice, which exhibited mildly reduced mitochondrial respiratory activity in COX/SDH staining, was comparable to that in G ≥ 10 and G1 *Polg* ^+/+^ mice, respectively (Figure S7). Additionally, the frequency of small deletions and insertions in G1 *Polg* mice was lower than that in G ≥ 10 *Polg* mice (Figure S7A,B). For example, the frequency of small deletions in G1 *Polg* ^mut/mut^ mice was approximately 0.01%, which was comparable to that in G ≥ 10 *Polg* ^+/mut^ mice (Figure S7A,B). However, no individuals in the G ≥ 10 *Polg* ^+/mut^ group exhibited severely reduced mitochondrial respiratory activity, similar to that observed in G1 *Polg* ^mut/mut^ mice, as assessed by COX/SDH staining (Figures S1A–C and S6A–C). Furthermore, the frequency of small deletions and insertions in G ≥ 10 and G1 *Polg* mice was more than 40 times lower than that of point mutations (Figure S7A,B). These findings suggest that point mutations in mtDNA have a greater impact on mitochondrial respiratory function than small deletions and insertions.

### 3.7 Reduced Mitochondrial Respiratory Activity Observed in *Polg* Mice Is Correlated With the Nuclear Genotype of *Polg*

**FIGURE 6:**
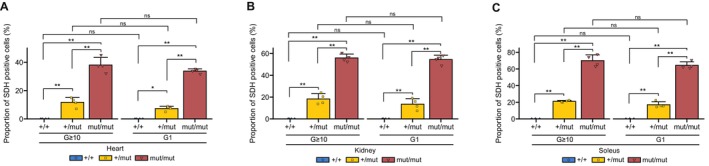
Quantitative COX/SDH staining results for G ≥ 10 and G1 *Polg* mice. (A–C) Quantitative COX/SDH staining results for the (A) heart, (B) kidney, and (C) soleus of G ≥ 10 and G1 *Polg* mice (*n* = 4, each group). The y‐axis represents the percentage of SDH‐positive cells among the total number of cells counted. Data are reported as mean ± SD. *p < 0.05, **p < 0.01 (Tukey–Kramer test).

Finally, to investigate the correlation between the nuclear genotype of *Polg* and mitochondrial respiratory activity, COX/SDH staining results from the organs of G ≥ 10 and G1 *Polg* mice were quantified (Figure 6A–C). The results revealed a correlation between the nuclear genotype of *Polg* and the proportion of cells with reduced mitochondrial respiratory activity in the organs of G1 and G ≥ 10 *Polg* mice, with significant differences across all genotypes (Figure 6A–C). Furthermore, despite variations in mtDNA genotype between G ≥ 10 and G1 *Polg* mice (Figures 1A, 2B, 3A,C), no significant differences were observed in quantitative COX/SDH staining results (Figure 6A–C).

Overall, these findings indicate that reduced mitochondrial respiratory activity in *Polg* mice is closely associated with the nuclear genotype of *Polg* rather than the mtDNA genotype.

## 4 Discussion

Previous studies have suggested that the accumulation of random point mutations in mtDNA, particularly non‐synonymous substitutions, leads to mitochondrial respiratory dysfunction and have mostly made comparisons between *Polg* ^+/+^ and *Polg* ^mut/mut^ mice (Edgar et al. 2009; Trifunovic et al. 2004). In contrast, a study focusing on *Polg* ^+/mut^ mice suggested that the accumulation of random point mutations in mtDNA did not induce aging (Vermulst et al. 2007). However, these previous studies have not examined the correlation between mtDNA genotype and mitochondrial respiratory dysfunction in *Polg* mice, including *Polg* ^+/mut^ mice. In this study, mtDNA genotype was determined in various organs of *Polg* mice using NGS analysis, and their correlation with mitochondrial respiratory activity was assessed using COX/SDH staining. The present study showed that compared with G ≥ 10 *Polg* ^+/+^ mice, G ≥ 10 *Polg* ^mut/mut^ mice exhibited significantly increased mtDNA mutation frequency (Figures 1A and S7A) and a higher number of non‐synonymous substitutions (Figure 3A), along with reduced mitochondrial respiratory activity (Figure 6A–C). These findings, consistent with previous research (Edgar et al. 2009; Trifunovic et al. 2004), reinforce the hypothesis that random point mutations and non‐synonymous substitutions in mtDNA contribute to mitochondrial dysfunction. However, G ≥ 10 *Polg* ^+/mut^ mice exhibited mildly reduced mitochondrial activity despite having a similar mtDNA genotype to those of G > 10 *Polg* ^+/+^ mice (Figures 1C, 2A, 3A,C). Since this result suggests that the reduced mitochondrial respiratory activity in *Polg* mice was not caused by the accumulation of random point mutations or small deletions and insertions in mtDNA, we generated G1 *Polg* mice, which have a lower mutation frequency compared to G ≥ 10 *Polg* mice (Figures 1A, 2A,B, S7A,B). Notably, even when the mutation frequency was reduced, mitochondrial respiratory activity did not improve (Figure 4A). Additionally, mitochondrial respiratory activity was severely reduced in G1 *Polg* ^mut/mut^ mice despite having mutation frequency and non‐synonymous substitutions similar to G ≥ 10 *Polg* ^+/+^ mice (Figures 4B and 5A). Moreover, the number of pathogenic mutations in G1 *Polg* ^mut/mut^ mice was the same as that in G1 *Polg* ^+/+^ mice (Figure 5C). Collectively, these results suggest that the accumulation of random point mutations and small deletions and insertions in mtDNA, i.e., mtDNA genotype, may not induce mitochondrial respiratory dysfunction in *Polg* ^mut/mut^ mice.

A previous study suggested that non‐synonymous substitutions occurring in mtDNA are less likely to be maternally transmitted due to purifying selection in *Polg* mice (Stewart et al. 2008). However, our study demonstrated that G ≥ 10 *Polg* mice accumulated more non‐synonymous substitutions than G1 *Polg* mice (Figure 3A,B). This discrepancy may be attributed to the presence of the proofreading‐deficient *Polg* allele in female mice during mating. The previous study tested germline selection pressure by crossing wild‐type male mice with wild‐type *Polg* females carrying maternally inherited mtDNA mutations randomly accumulated in *Polg* ^mut/mut^ mice (Stewart et al. 2008). In this context, no new non‐synonymous substitutions were expected to occur in mtDNA due to the presence of proofreading‐deficient *Polg* in the female germline (Stewart et al. 2008). However, in this study, the maintenance of mouse strains by mating *Polg* ^+/mut^ mice always made the mtDNA molecule in female mouse germ cells prone to new non‐synonymous substitutions through proofreading‐deficient mutations in *Polg*. Consequently, non‐synonymous substitutions may have been maternally inherited by subsequent generations beyond the purifying selection observed in prior studies. This allowed us to explore the causal relationship between the accumulation of non‐synonymous substitutions in mtDNA and reduced mitochondrial respiratory function in *Polg* mice.

When pathogenic mutations accumulate over a threshold in mtDNA molecules, mitochondrial respiratory function declines and can be detected as COX‐/SDH+ cells in COX/SDH staining (Baines et al. 2014; Greaves et al. 2010). Previous research has indicated that mtDNA molecules with low‐frequency pathogenic mutations clonally expand over a threshold with age, resulting in mitochondrial respiratory dysfunction in *Polg* ^mut/mut^ mice (Trifunovic et al. 2004; Vermulst et al. 2008). In the present study, COX‐/SDH+ cells were identified in *Polg* ^+/mut^ and *Polg* ^mut/mut^ mice (Figure 5A–C). If non‐synonymous substitutions and pathogenic mutations clonally accumulate in COX‐negative cells, these mutations should be detected at low mutation frequencies in NGS results from bulk tissue. For example, in the heart of a G1 *Polg* ^mut/mut^ mouse, approximately 38% of cells were COX‐/SDH+ (Figure 6A), and if these COX‐/SDH+ cells had accumulated 50% of certain non‐synonymous substitutions and pathogenic mutations, the mutation frequency would be detected as approximately 19% (= 50% × 38%) in the NGS analysis from the bulk tissue. In other words, if the clonal accumulation of non‐synonymous substitutions and pathogenic mutations occurs in COX‐/SDH+ cells in *Polg* ^mut/mut^ mice, the number of non‐synonymous substitutions and pathogenic mutations with low mutation frequency should be higher in *Polg* ^mut/mut^ mice than in *Polg* ^+/+^ mice. Therefore, the number of non‐synonymous substitutions and pathogenic mutations with low mutation frequency (> 1%) was examined in relation to mitochondrial respiratory activity (COX‐/SDH+ staining). The results showed that G ≥ 10 *Polg* ^+/+^ mice had a similar number of non‐synonymous substitutions as G1 *Polg* ^mut/mut^ mice, in which COX‐/SDH+ cells were observed, but no COX‐/SDH+ cells were identified in the former (Figure 5A). Similarly, G1 *Polg* ^+/+^ mice exhibited a comparable number of pathogenic mutations to G1 *Polg* ^mut/mut^ mice, but no COX‐/SDH+ cells were identified (Figure 5C). These findings suggest that the COX‐/SDH+ cells observed in *Polg* ^mut/mut^ mice are not due to clonal accumulation of non‐synonymous substitutions or pathogenic mutations, at least at a mutation frequency of 1%.

In previous studies, PCR‐amplified fragments of mtDNA were used to analyze mtDNA mutation frequency in *Polg* mice (Kujoth et al. 2005; Trifunovic et al. 2004; Vermulst et al. 2007). In this study, next‐generation sequencing was conducted to accurately detect mtDNA mutations using enriched circular mtDNA without PCR amplification, as referenced in prior research (Bagge et al. 2020). The findings, consistent with previous studies, demonstrated that significant accumulation of mtDNA mutations occurred in all organs of G1 and G ≥ 10 *Polg* ^mut/mut^ mice compared with G1 and G ≥ 10 *Polg* ^+/+^ mice, although the mutation frequency was at most 1.7 times higher than that in *Polg* ^+/+^ mice (Figures 1A and 2B). Previous studies reported that compared with those in *Polg* ^+/+^ mice, a mutation frequency in *Polg* ^mut/mut^ mice could increase 3‐8‐fold (Kujoth et al. 2005; Trifunovic et al. 2004) or up to 2500‐fold (Vermulst et al. 2007). In contrast, the relative mutation frequency calculated in this study was lower (Figures 1A and 2B). The proofreading function of *Polg*, through its exonuclease activity, facilitates ligation during mtDNA replication (Macao et al. 2015) and degrades fragmented mtDNA (Nissanka et al. 2018; Peeva et al. 2018). Consequently, fragmented linear mtDNA has been observed to increase in cells and mice with *Polg*‐deficient proofreading (Nissanka et al. 2018; Peeva et al. 2018; Trifunovic et al. 2004). NUMTs, sequences within nuclear DNA that resemble mtDNA, may be misidentified during mtDNA sequencing (Goios et al. 2009; Hazkani‐Covo et al. 2010). Therefore, previous studies that relied on sequencing PCR‐amplified fragments of specific regions or the full length of mtDNA may have amplified fragmented linear mtDNA and NUMTs. However, no significant increase in NUMTs has been reported in *Polg* ^mut/mut^ mice. Hence, previous studies may have overestimated mutation frequency in *Polg* ^mut/mut^ mice by including fragmented linear mtDNA in their sequencing data.

Mitochondrial respiratory dysfunction is one of the contributing factors to aging (López‐Otín et al. 2023; Wallace 1999). *Polg* ^mut/mut^ mice exhibit severely reduced mitochondrial respiratory activity (Figures 1C, 4A,B and 6A–C) and premature aging phenotypes (Kujoth et al. 2005; Trifunovic et al. 2004). Conversely, mice with pathogenic mutations in mtDNA show reduced mitochondrial respiratory function but mitochondrial diseases and diseases associated with mitochondria rather than a premature aging phenotype (Fan et al. 2008; Hashizume et al. 2012; Inoue et al. 2000; Kasahara et al. 2006; Lin et al. 2012; Shimizu et al. 2014; Tani et al. 2022; Tyynismaa et al. 2005; Yokota et al. 2010; Zhang et al. 2025). This raises the question of why *Polg* ^mut/mut^ mice exhibit a premature aging phenotype, whereas mice with pathogenic mutations in mtDNA exhibit mitochondrial diseases despite both exhibiting mitochondrial respiratory dysfunction. Previous studies have shown that *Polg* ^mut/mut^ mice exhibit somatic stem cell dysfunction (Ahlqvist et al. 2012) and immunometabolism dysfunction (Lei et al. 2021), which could be key driving forces behind premature aging phenotypes. Therefore, to understand the mechanisms underlying premature aging in *Polg* ^mut/mut^ mice, it will be necessary to investigate various aspects of the aging process beyond mitochondrial respiratory dysfunction.

In this study, G ≥ 10 and G1 *Polg* ^+/mut^ mice exhibited a similar mtDNA mutation frequency as G ≥ 10 and G1 *Polg* ^+/+^ mice, respectively, despite carrying a proofreading‐deficient mutation in the *Polg* gene (Figures 1A and 2B). The reason for this remains unclear. One possibility is that, in *Polg* ^+/mut^ mice, mutant mtDNA is selectively degraded through mechanisms such as mitophagy. Alternatively, only mutations that can be repaired may have occurred. Future studies should focus on *Polg* ^+/mut^ mice to elucidate the mechanisms that prevent the accumulation of mtDNA mutations in these mice.

In conclusion, this study provides evidence that mitochondrial respiratory dysfunction in *Polg* ^mut/mut^ mice is more likely associated with the nuclear genotype of the *Polg* gene rather than the accumulation of point mutations in mtDNA (Figure 6A–C). Previous findings have suggested that the exonuclease activity of *Polg* may play roles beyond mtDNA proofreading, including the degradation of linear mtDNA fragments (Nissanka et al. 2018; Peeva et al. 2018) and the ligation process during mtDNA replication (Macao et al. 2015). Additionally, studies have suggested the interactions between POLG and other factors unrelated to mtDNA replication and proofreading (Liyanage et al. 2017). Thus, the reduced mitochondrial respiratory function in *Polg* ^mut/mut^ mice may be caused by a disruption in unknown functions of *Polg*. Therefore, it is crucial to reconsider the mitochondrial theory of aging in *Polg* ^mut/mut^ mice with a focus on these unidentified functions of *Polg*.

## Author Contributions

**Hiroaki Tamashiro:** writing – original draft, writing – review and editing, investigation, data curation, visualization, methodology, resources, formal analysis, funding acquisition. **Kaori Ishikawa:** writing – original draft, writing – review and editing, investigation, validation, resources, methodology, funding acquisition. **Koichi Sadotomo:** resources. **Emi Ogasawara:** investigation, resources, funding acquisition. **Kazuto Nakada:** writing – original draft, writing – review and editing, funding acquisition, conceptualization, project administration, supervision.

## Conflicts of Interest

The authors declare no conflicts of interest.

## Supporting information


**Figure S1.** Mitochondrial respiratory activity, mtDNA mutation frequency, non‐synonymous substitutions, and pathogenic mutations in G ≥ 10 *Polg* mice.

**Figure S2.** mtDNA mutation frequency in organs of G ≥ 10 *Polg* ^+/+^, *Polg* ^+/mut^, and *Polg* ^mut/mut^ mice.

**Figure S3.** Mutation frequency of mtDNA bases in the organs of G1 *Polg* ^+/+^, *Polg* ^+/mut^, and *Polg* ^mut/mut^ mice.

**Figure S4.** Mutation frequency of non‐synonymous substitutions in the organs of G ≥ 10 and G1 *Polg* mice.

**Figure S5.** Mutation frequency of pathogenic mutations in the organs of G ≥ 10 and G1 *Polg* mice.

**Figure S6.** Mitochondrial respiratory activity, mtDNA mutation frequency, non‐synonymous substitutions, and pathogenic mutations in G1 *Polg* mice.

**Figure S7.** Small insertion and deletion frequency in G ≥ 10 and G1 *Polg* mice.

**Table S1.** Total read numbers, depth and coverage rate for all samples analyzed by NGS in this study.

## Data Availability

Sequencing data has been deposited to the Sequencing Read Archive at NCBI under accession number PRJNA1195219 (https://dataview.ncbi.nlm.nih.gov/object/PRJNA1195219?reviewer=lvlgfilldugpen0bt0bjd982em). Any additional information required to reanalyze the data reported in this paper is available from the lead contact upon request.
