# Supplementary figures and images for: Mitochondrial Respiratory Dysfunction Is Not Correlated With Mitochondrial Genotype in Premature Aging Mice
Source: Aging Cell. 2025 May 2;24(7):e70085. doi: 10.1111/acel.70085 (PMC12266765; doi:10.1111/acel.70085)

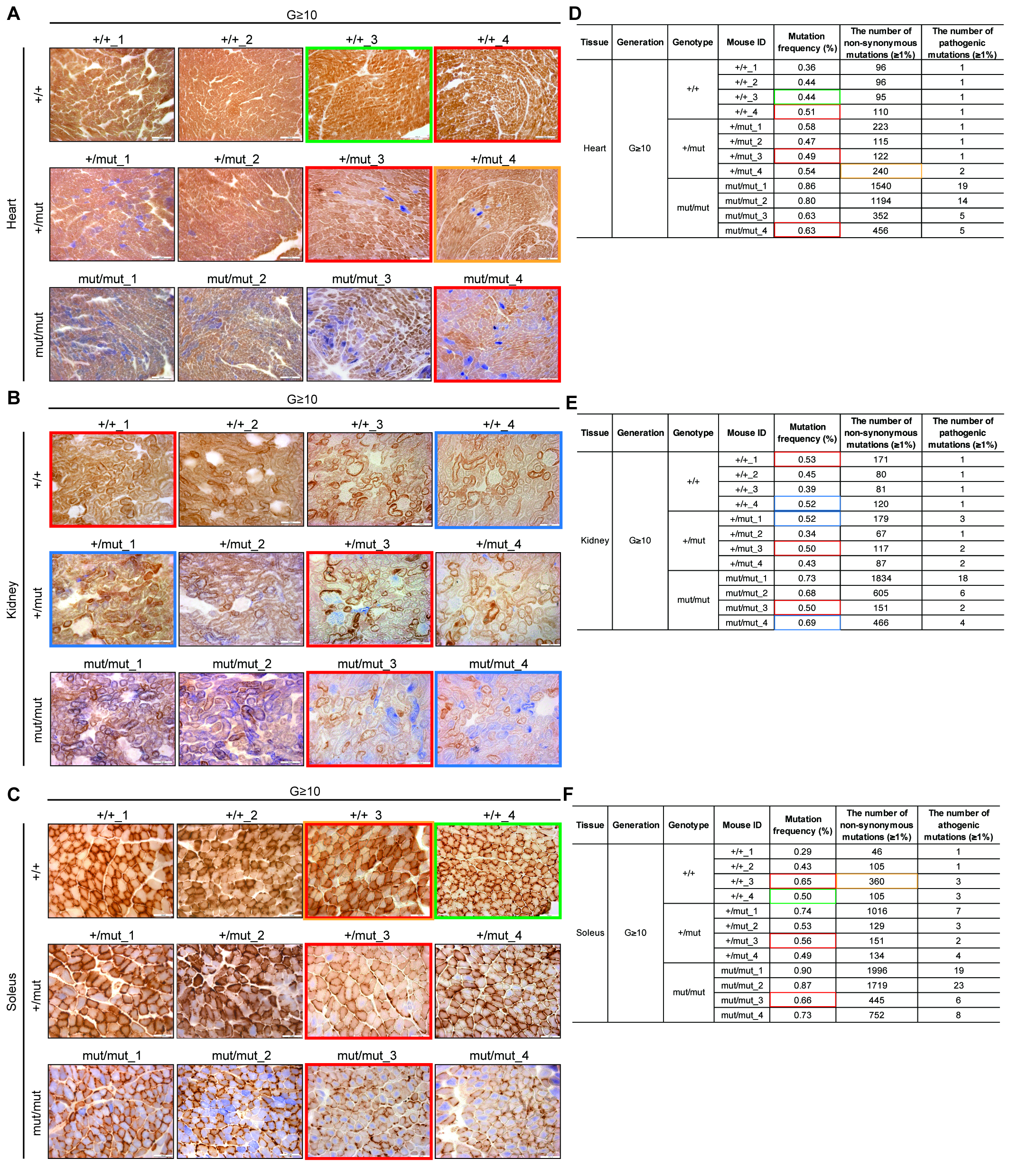

Supplement: Supplementary file 1 — Figure S1. Mitochondrial respiratory activity, mtDNA mutation frequency, non‐synonymous substitutions, and pathogenic mutations in G ≥ 10 Polg mice. [file ACEL-24-e70085-s007.tif]

**A**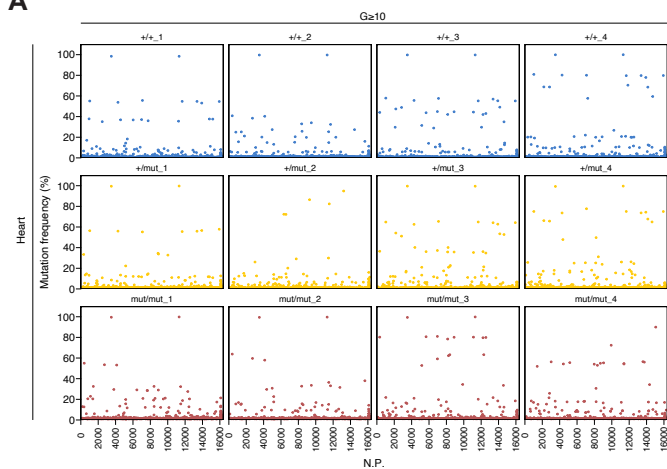**B**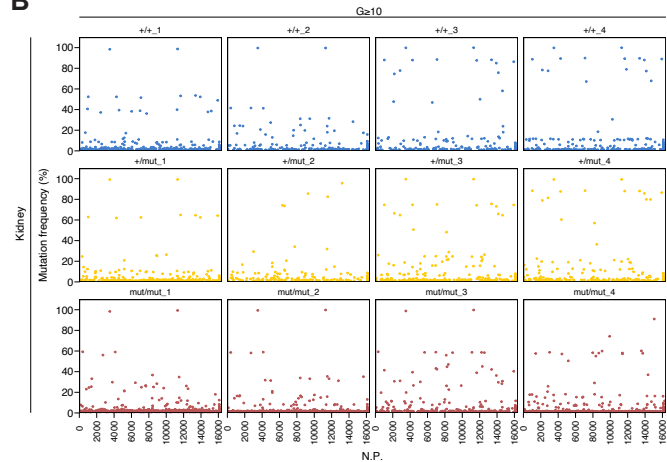**C**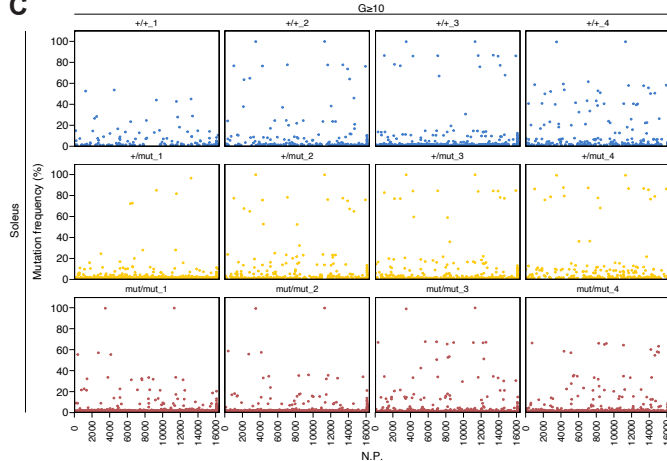**D**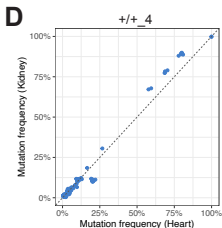**E**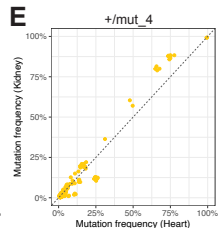**F**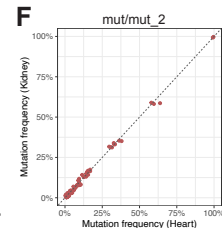**G**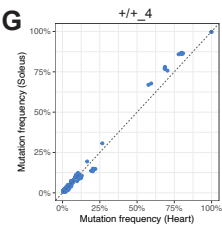**H**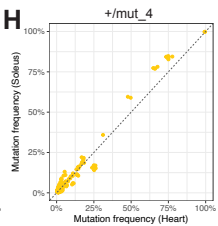**I**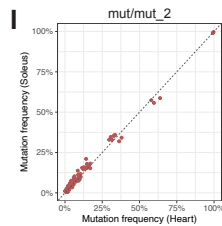

Supplement: Supplementary file 2 — Figure S2. mtDNA mutation frequency in organs of G ≥ 10 Polg +/+, Polg +/mut, and Polg mut/mut mice. [file ACEL-24-e70085-s008.pdf]

**A**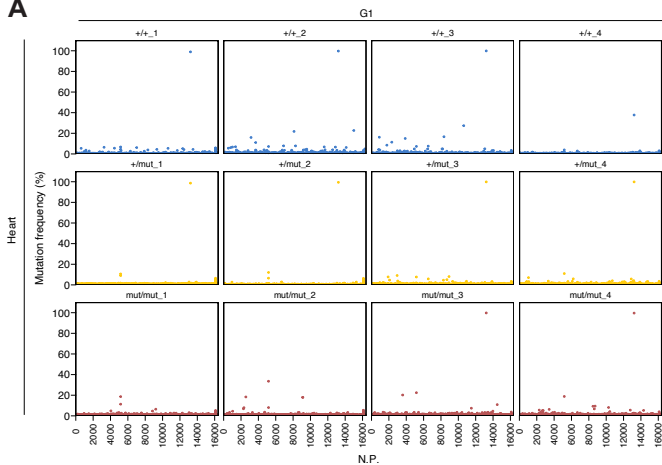**B**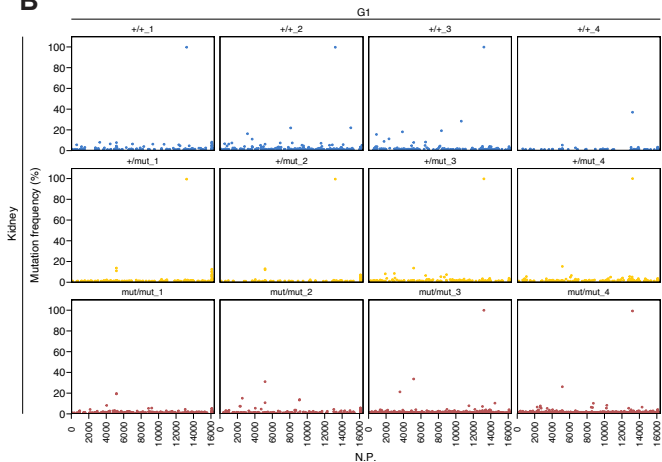**C**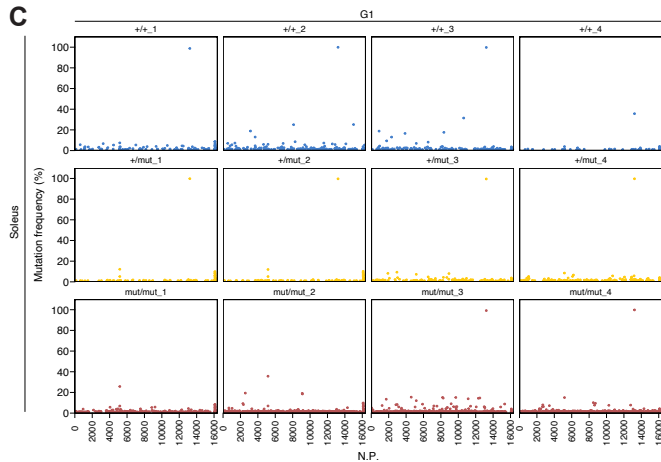

Supplement: Supplementary file 3 — Figure S3. Mutation frequency of mtDNA bases in the organs of G1 Polg +/+, Polg +/mut, and Polg mut/mut mice. [file ACEL-24-e70085-s004.pdf]

**A**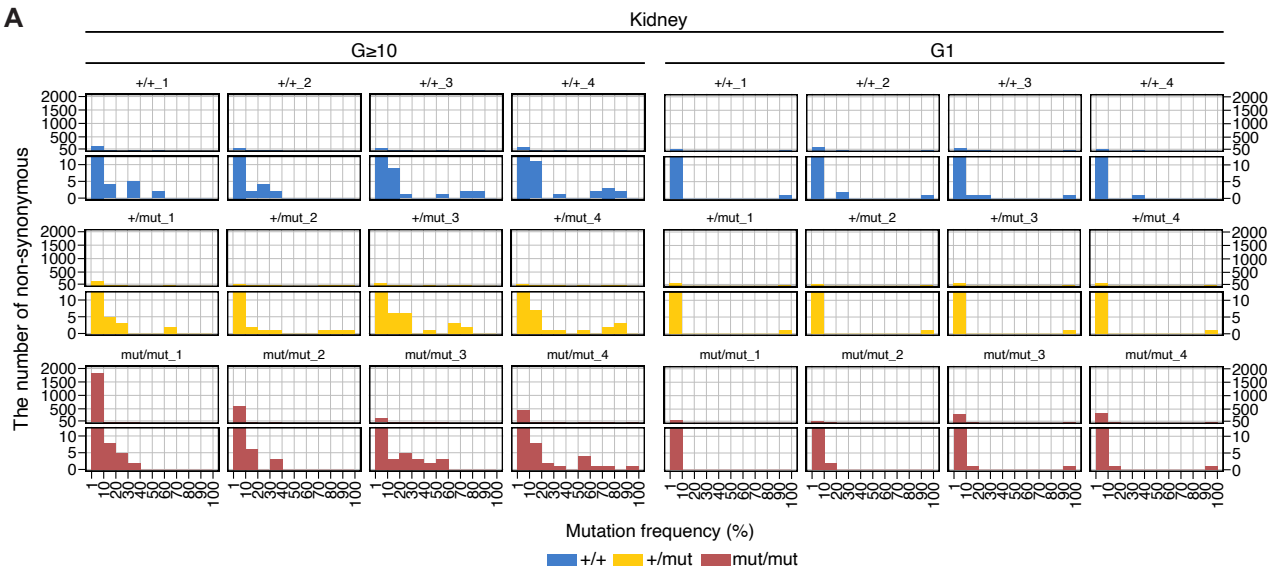**B**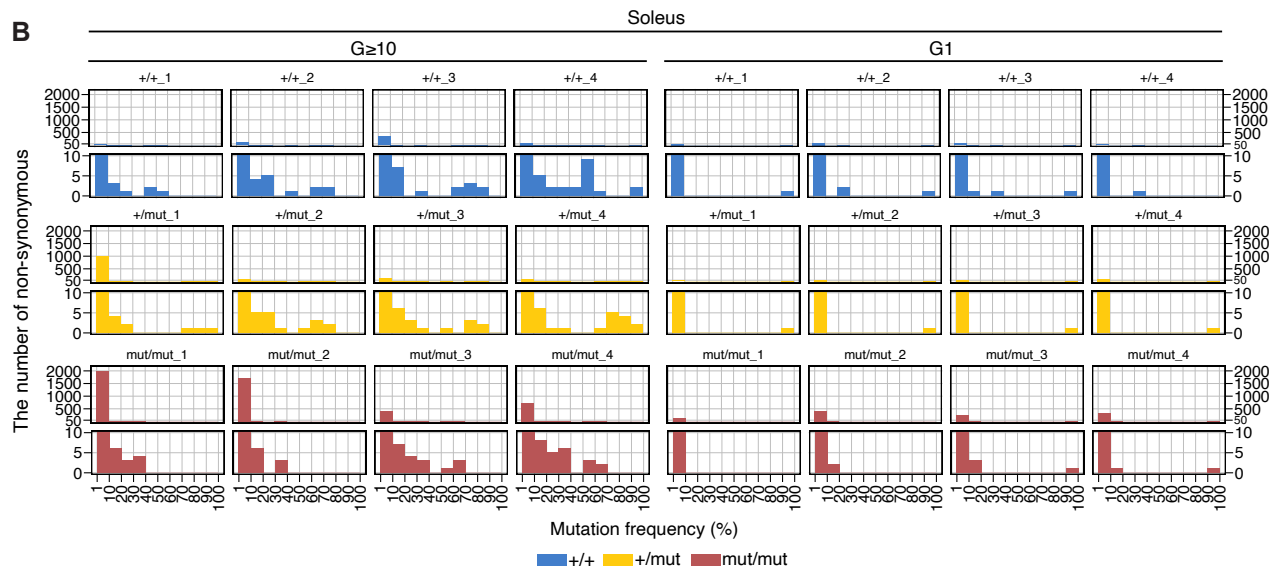

Supplement: Supplementary file 4 — Figure S4. Mutation frequency of non‐synonymous substitutions in the organs of G ≥ 10 and G1 Polg mice. [file ACEL-24-e70085-s001.pdf]

**A**

Kidney

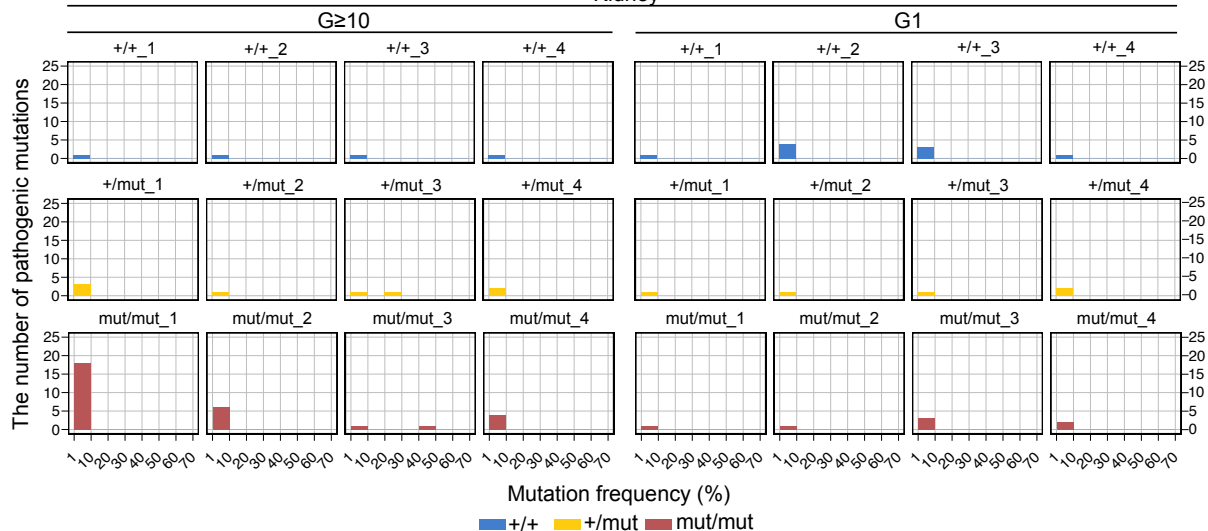**B**

Soleus

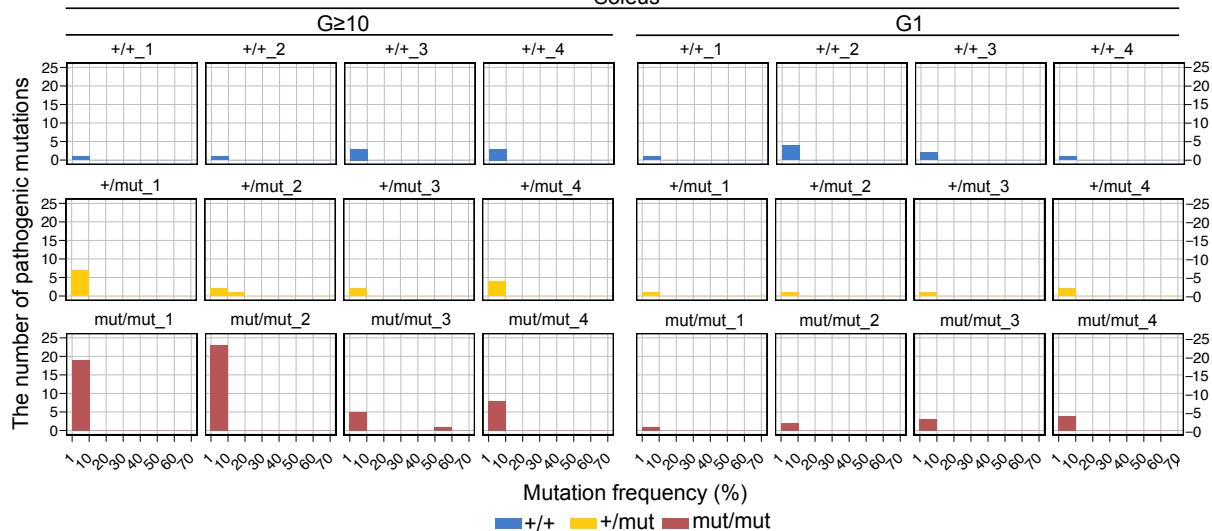

Supplement: Supplementary file 5 — Figure S5. Mutation frequency of pathogenic mutations in the organs of G ≥ 10 and G1 Polg mice. [file ACEL-24-e70085-s006.pdf]

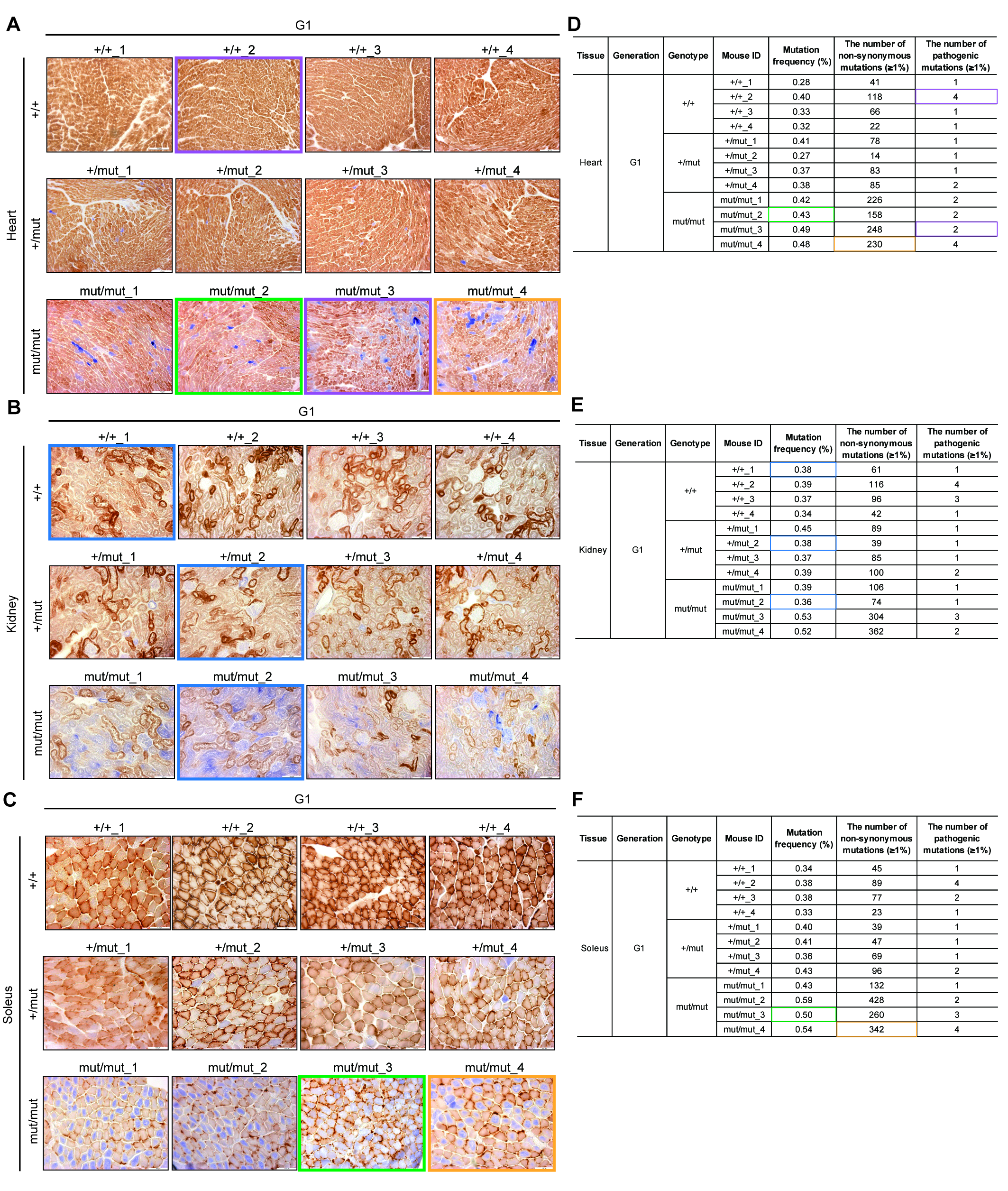

Supplement: Supplementary file 6 — Figure S6. Mitochondrial respiratory activity, mtDNA mutation frequency, non‐synonymous substitutions, and pathogenic mutations in G1 Polg mice. [file ACEL-24-e70085-s003.tif]

**A**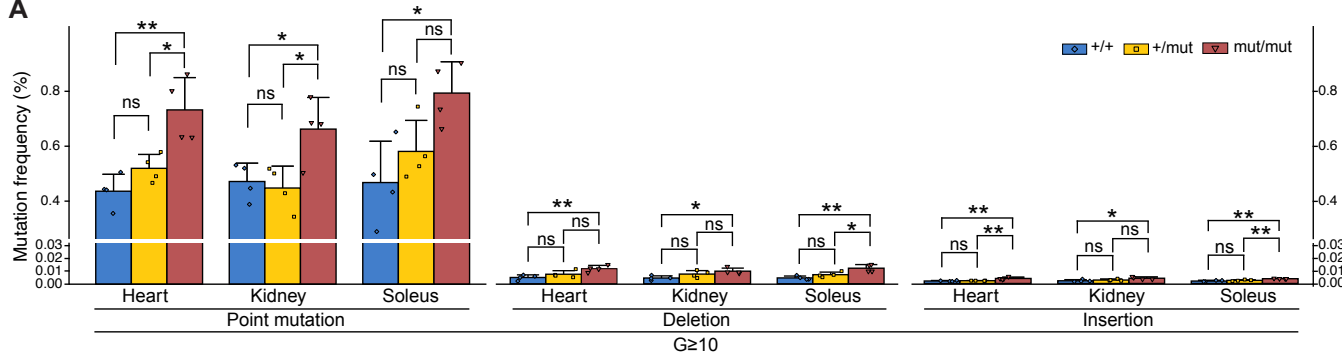**B**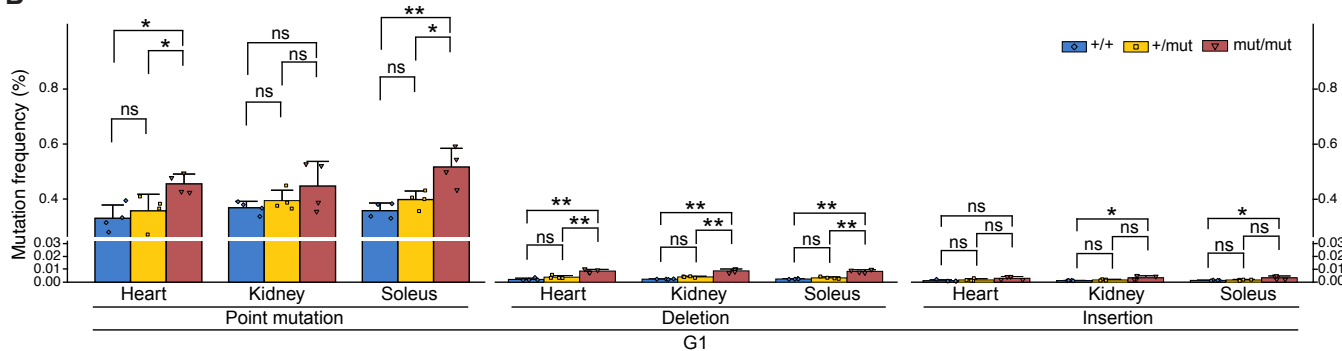

Supplement: Supplementary file 7 — Figure S7. Small insertion and deletion frequency in G ≥ 10 and G1 Polg mice. [file ACEL-24-e70085-s002.pdf]
